# Healthcare utilization 9 months pre- and post- COVID-19 hospitalization among patients discharged alive

**DOI:** 10.1371/journal.pone.0303509

**Published:** 2024-06-20

**Authors:** Mohammed Zaidan, Daniel Puebla Neira, Efstathia Polychronopoulou, Kuo Yong-Fang, Gulshan Sharma

**Affiliations:** 1 Department of Internal Medicine, Division of Pulmonary, Critical Care and Sleep Medicine, University of Texas Medical Branch (UTMB), Galveston, TX, United States of America; 2 Department of Internal Medicine, Division of Pulmonary, Critical Care and Sleep Medicine, University of Arizona College of Medicine, Phoenix, AZ, United States of America; 3 Office of Biostatistics, University of Texas Medical Branch (UTMB), Galveston, TX, United States of America; 4 Sealy Center on Aging, University of Texas Medical Branch (UTMB), Galveston, TX, United States of America; Stanford University School of Medicine, UNITED STATES

## Abstract

**Background:**

Emerging evidence suggests that there is an increase in healthcare utilization (HCU) in patients due to Coronavirus Disease 2019 (COVID-19). We investigated the change in HCU pre and post hospitalization among patients discharged home from COVID-19 hospitalization for up to 9 months of follow up.

**Study design and methods:**

This retrospective study from a United States cohort used Optum® de-identified Clinformatics Data Mart; it included adults discharged home post hospitalization with primary diagnosis of COVID-19 between April 2020 and March 2021. We evaluated HCU of patients 9 months pre and post -discharge from index hospitalization. We defined HCU as emergency department (ED), inpatient, outpatient (office), rehabilitation/skilled nursing facility (SNF), telemedicine visits, and length of stay, expressed as number of visits per 10,000 person-days.

**Results:**

We identified 63,161 patients discharged home after COVID-19 hospitalization. The cohort of patients was mostly white (58.8%) and women (53.7%), with mean age 72.4 (SD± 12) years. These patients were significantly more likely to have increased HCU in the 9 months post hospitalization compared to the 9 months prior. Patients had a 47%, 67%, 65%, and 51% increased risk of ED (rate ratio 1.47; 95% CI 1.45–1.49; p < .0001), rehabilitation (rate ratio 1.67; 95% CI 1.61–1.73; p < .0001), office (rate ratio1.65; 95% CI 1.64–1.65; p < .0001), and telemedicine visits (rate ratio 1.5; 95% CI 1.48–1.54; p < .0001), respectively. We also found significantly different rates of HCU for women compared to men (women have higher risk of ED, rehabilitation, and telemedicine visits but a lower risk of inpatient visits, length of stay, and office visits than men) and for patients who received care in the intensive care unit (ICU) vs those who did not (ICU patients had increased risk of ED, inpatient, office, and telemedicine visits and longer length of stay but a lower risk of rehabilitation visits). Outpatient (office) visits were the highest healthcare service utilized post discharge (64.5% increase). Finally, the risk of having an outpatient visit to any of the specialties studied significantly increased post discharge. Interestingly, the risk of requiring a visit to pulmonary medicine was the highest amongst the specialties studied (rate ratio 3.35, 95% CI 3.26–3.45, p < .0001).

**Conclusion:**

HCU was higher after index hospitalization compared to 9 months prior among patients discharged home post-COVID-19 hospitalization. The increases in HCU may be driven by those patients who received care in the ICU.

## Introduction

Most patients are discharged alive from hospitalization due to Coronavirus Disease 2019 (COVID-19). With over 6 million COVID-19 hospitalizations in the United States (US), there is growing concern regarding the health care utilization (HCU) of patients post discharge [1–4]. Prior reports of non-COVID-19 patients show high HCU post hospitalization and post care in an intensive care unit (ICU) [5–8]. Based on this evidence, HCU is expected to be high for patients discharged from COVID-19 admission.

Overall, studies have found that patients who tested positive for severe acute respiratory syndrome coronavirus-2 (SARS-COV-2) have greater HCU than patients who tested negative [9, 10]. Also, the 30-day and 60-day readmission rate for patients discharged alive after a hospitalization due to COVID has been reported as 13% to 24% and 19.9%, respectively [2, 11–17]. Similarly, the rates of ambulatory visits, emergency department (ED) visits, and hospitalizations vary following discharge from COVID-19 in socially disadvantaged patients compared to those who are socially advantaged [18, 19]. It has been reported that 82.1% of patients had follow-up visits with a primary care provider in the 60 days following discharge from COVID-19 hospitalization [20]. However, these observations were obtained from cohorts of patients who were ambulatory and/or post-hospital discharge. The HCU of patients discharged home post-COVID-19 hospitalization is not well understood. We, therefore, investigated the HCU of patients discharged home from COVID-19 hospitalization for 9 months post-discharge, using a national US database. We hypothesized that HCU will be high post discharge and that certain specialties will see a disproportionate increase in their share of post-discharge HCU. Some of the results of this study were previously presented in the form of a conference abstract [21].

## Study design and methods

### Data source

We used de-identified data from Optum’s Clinformatics Data Mart (CDM), a database of administrative health claims for members of large commercial and Medicare Advantage health plans. Optum’s CDM is a comprehensive database that includes claims from enrollees with either commercial insurance or Medicare Advantage Plans, encompassing over 63 million unique enrollees across the United States from all states. Notably, more than 95% of the enrollees in Optum’s CDM possess commercial insurance. This database does not include traditional Medicare or Medicaid enrollees, implying a potential underrepresentation of the older or low socioeconomic status populations. A significant aspect of the data from Optum’s CDM is its source diversity; since the data originates from insurance claims. This inclusivity means data can come from a variety of healthcare settings, whether rural or urban, academic or community-based. Whenever a CDM patient files a claim with their insurance, this information is captured in the database, regardless of the specific medical center or office they visited. This wide-ranging data collection provides a broad and diverse view of healthcare utilization and patterns across different demographics and geographies in the United States. The University of Texas Medical Branch Institutional Review Board (IRB) approved this study (IRB# 20–0180). The need for informed consent was waived by the IRB due to the de-identified nature of the study.

### Cohort selection

**Fig 1:**
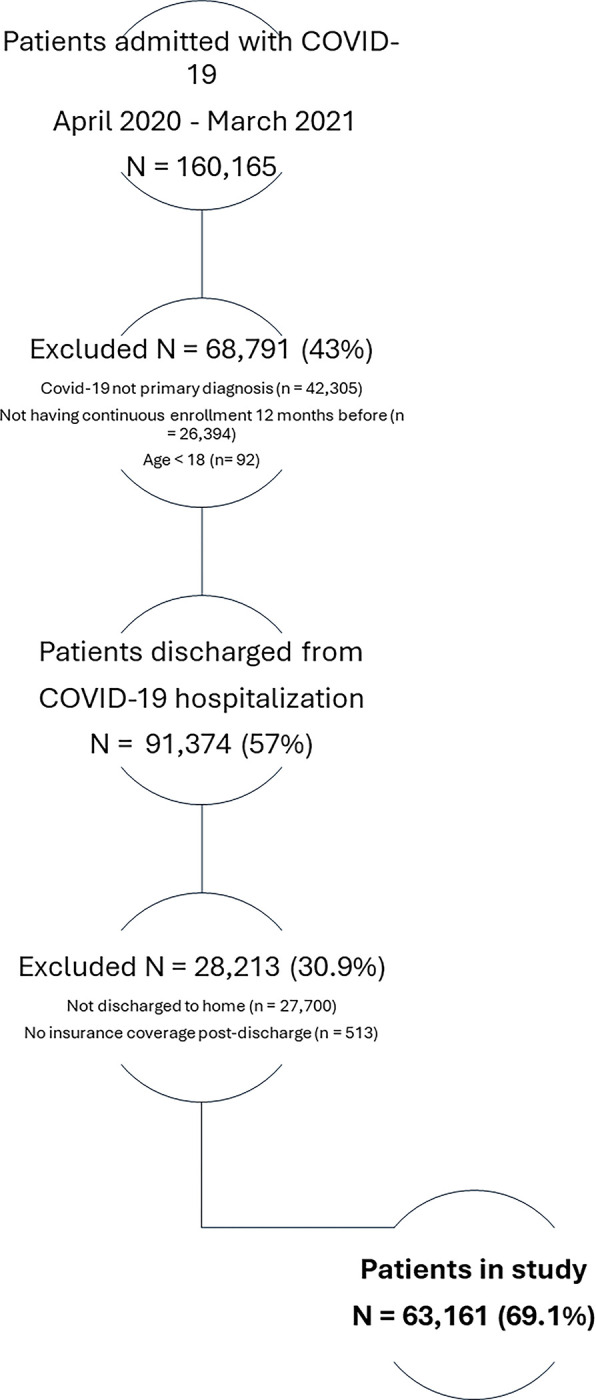
Cohort selection of patients discharged from COVID-19 hospitalization. COVID-19 was identified by the ICD-10-CM diagnosis code U07.1 or from a positive test. Patients were excluded if they were not discharged home or if their insurance coverage ended during the inpatient stay. *Abbreviations*: COVID-19: Coronavirus Disease 2019; Optum CDM: Optum’s Clinformatics Data Mart. ICD-10-CM: International Classification for Diseases, tenth revision, clinical modification.

Our sample included all adults hospitalized with a primary diagnosis of COVID-19 who were discharged home (with or without home health care) between April 2020 and March 2021, with at least 12 months of continuous enrollment before this diagnosis. COVID-19 cases were identified by the International Classification for Diseases, tenth revision, clinical modification (ICD-10-CM) diagnosis code U07.1 or from a positive test. We excluded patients who were not discharged home or whose insurance coverage ended during the inpatient stay (Fig 1).

### Variables

We collected information on patient demographics: age at time of COVID-19 diagnosis, sex, race/ethnicity, and region of residence. We identified comorbidities by ICD-10-CM diagnosis codes, with a 12-month lookback period before COVID-19 hospitalization.

We evaluated HCU in the 9 months pre and post hospitalization from COVID-19. We divided the study period into pre-COVID-19 hospitalization (-0-9 months) and post-COVID-19 discharge (+0–9 months). We also subdivided into 3-month periods for pre-COVID-19 hospitalization (-0-3 months, -3-6 months, and -6-9 months before hospitalization) and post-COVID-19 discharge (+0–3 months, +3–6 months, and +6–9 months post discharge). HCU included ED visits, inpatient admissions, rehabilitation/skilled nursing facility (SNF) admissions, outpatient visits, and telemedicine visits. Based on billing information, we examined outpatient visits (from here on out referred to as office visits) to primary care providers, including family medicine, internal medicine, and nurse practitioner visits, and certain sub-specialties, such as cardiology, pulmonary medicine, endocrinology, neurology, physical medicine and rehabilitation, psychiatry, and other mental health professionals.

### Statistical analysis

Cohort characteristics are presented as mean and standard deviation (SD), median and interquartile range, or frequency and percentage. HCU in the pre-COVID-19 hospitalization and post-COVID-19 discharge periods was expressed as the number of visits per 10,000 (10k) person-days, where each patient contributed person-days until death, loss of insurance eligibility, or the end of the 9-month follow-up period. To identify the risk of HCU post-COVID-19 compared to pre-COVID-19 hospitalization incidence rate ratios (number of observed events over total person-days contributed) and exact Poisson confidence intervals were calculated for each period [22]. All findings were considered statistically significant if the *P* value was <0.05. To address the concern of survivor bias, the study required participants to have at least 12 months of data prior to their primary COVID-related hospitalization for accurate comorbidity assessment, without mandating post-hospitalization follow-up, and utilized a person-days approach to accommodate variable follow-up durations. As a sensitivity analysis, we evaluated HCU post discharge in women and compared it to that of men. In addition, we analyzed HCU in patients who received care in the ICU and compared it to those who did not. We also examined the distribution of office visits per 10K person-days by provider specialty (select specialties) in each period and calculated rate ratios to identify the risk of having a visit with that specialist post-COVID-19 hospitalization compared to the pre-hospitalization period. All analyses were conducted using SAS version 9.2 (SAS Institute, Inc., Cary NC).

## Results

In this retrospective study, we identified 63,161 patients discharged home after hospitalization due to COVID-19. This cohort was comprised of patients who were mostly white (58.8%), women (53.7%), and with mean age of 72.4 (SD± 12.8) years. The most common comorbidities of these patients were hypertension (75.8%), diabetes mellitus (36.5%), congestive heart failure (25.8%), coronary artery disease (25.2%), and chronic obstructive pulmonary disease (23%) (Table 1).

**Table 1:** Characteristics of patients discharged home from COVID-19 hospitalization from April 2020 to March 2021 in the United States.

Characteristics	N = 63,161 (100%)^a^^,^^b^^,^^c^
**Age, mean (SD) **	72.4 (12.8)
**Sex **	-
Women	33897 (53.7)
Men	29263 (46.3)
**Race/Ethnicity** ^**d**^^,^^**e**^	-
White	37135 (58.8)
Hispanic	9843 (15.6)
Black	10443 (16.5)
Asian	1473 (2.3)
Other/Unknown	4261 (6.8)
**Region **	-
Midwest	13897 (22)
Northeast	8465 (13.4)
South	30145 (47.7)
West	9299 (14.7)
Unknown	1355 (2.1)
**Comorbidities **	-
DM	23,065 (36.5)
HTN	47,890 (75.8)
Asthma	6,285 (10.0)
COPD	14,534 (23.0)
CKD	6,793 (10.8)
ESRD	2,032 (3.2)
Stroke	5,547 (8.8)
CHF	16,303 (25.8)
Cancer	7,982 (12.6)
CAD	15,925 (25.2)
Liver disease	3,871 (6.1)
**ICU use **	21760 (34.5)

^a^ Our cohort consists of 63,161 patients discharged home after hospitalization due to COVID-19.

^b^ COVID-19 and comorbidities were identified by ICD-10-CM diagnosis codes(S7 Table in S1 File).

^c^ Patients were excluded if they were not discharged home or if their insurance coverage ended during the inpatient stay.

^d^ Patients self-identifying as non-Hispanic ethnicity were categorized based on race (White, Black, Asian, other/unknown).

^e^ Patients self-identifying as Hispanic ethnicity were included in the Hispanic group regardless of race.

*Definition of abbreviations*: COVID-19: Coronavirus Disease 2019; SD: Standard Deviation; DM: Diabetes Mellitus; HTN: Hypertension; COPD: Chronic Obstructive Pulmonary Disease; CKD: Chronic Kidney Disease; ESRD: End-Stage Renal Disease; CHF: Congestive Heart Failure; CAD: Coronary Artery Disease; ICU: Intensive Care Unit; ICD-10-CM International Classification for Diseases, tenth revision, clinical modification.

### Health care utilization after discharge home from hospitalization due to COVID-19

Patients discharged home from COVID-19 hospitalization were significantly more likely to have increased HCU in the 9 months post hospitalization compared to the 9 months prior to such hospitalization (Tables 2, 3, S1-S6, S8 Tables in S1 File). For example, these patients had 47%, 67%, 65%, and 51% increased risk of ED (rate ratio 1.47; 95% CI 1.45–1.49; p < .0001), rehabilitation (rate ratio 1.67; 95% CI 1.61–1.73; p < .0001), office (rate ratio1.65; 95% CI 1.64–1.65; p < .0001), and telemedicine visits (rate ratio 1.5; 95% CI 1.48–1.54; p < .0001), respectively. Also, the post-discharge risk of inpatient visits (rate ratio 2.20; 95% CI 2.14–2.25; p < .0001) and longer length of stay (rate ratio 2.62; 95% CI 2.59–2.64; p < .0001) doubled compared to the pre-COVID-19 hospitalization period (Table 2 and S1 Table in S1 File).

**Table 2:** Health care utilization of patients pre and post-hospitalization due to COVID-19^,c,d,e^.

-	Pre-COVID-19 Hospitalization ^a^^,^^b^	Post-COVID-19 Hospitalization ^a^^,^^b^	-	-	-
-	-0-9 months	+0–9 months	Percent change ^c^	Rate ratio (95% CI)	p-value
**ED visits **	22.0	32.3	47.0	1.47 (1.45–1.49)	< .0001
**Inpatient visits **	7.0	15.4	119.6	2.20 (2.14–2.25)	< .0001
**Inpatient admission (LOS)**^**d**^	42.7	111.6	161.6	2.62 (2.59–2.64)	< .0001
**Rehabilitation visits **	3.3	5.5	66.7	1.67 (1.61–1.73)	< .0001
**Office visits **	156.5	257.5	64.5	1.65 (1.64–1.65)	< .0001
**Telemedicine visits **	11.2	17.0	51.3	1.51 (1.48–1.54)	< .0001

^a^ Our cohort consists of 63,161 patients discharged home after hospitalization due to COVID-19.

^b^ Health care utilization of patients, measured by number of visits per 10k person-days.

^c^ Percent change of HCU pre- and post- hospitalization due to COVID-19.

^d^ LOS was defined as the number of days of inpatient status after hospital admission.

*Definition of abbreviations*: COVID-19: Coronavirus Disease 2019; ED: Emergency Department; ICD-10-CM: International Classification for Diseases, tenth revision, clinical modification; LOS: length of stay. 10k = 10,000

**Table 3:** Health care utilization of patients pre and post hospitalization due to COVID-19^,^, by medical specialty.

	Pre-COVID-19 Hospitalization^a^^,^^b^	Post-COVID-19 Hospitalization^a,b^	-	-	-
	-0-9 months	+0–9 months	Percent change ^c^	Rate ratio (95% CI)	p-value
**PCP **	78.8	133.1	68.9	1.69 (1.67–1.70)	< .0001
**Cardiology **	12.2	21.9	79.2	1.79 (1.76–1.83)	< .0001
**Pulmonary Medicine **	3.8	12.9	235.5	3.35 (3.26–3.45)	< .0001
**Endocrinology **	2.3	3.7	63.8	1.64 (1.57–1.71)	< .0001
**Neurology **	3.0	4.5	51.6	1.51 (1.46–1.58)	< .0001
**Phys Med & Rehab **	2.0	2.5	25.8	1.26 (1.20–1.32)	< .0001
**Psychiatry **	1.9	2.7	41.4	1.41 (1.35–1.49)	< .0001
**Mental Health Professional **	0.8	1.3	66.1	1.66 (1.54–1.79)	< .0001

^a^ Our cohort consists of 63,161 patients discharged home after hospitalization due to COVID-19.

^b^ Health care utilization of patients, measured by number of visits per 10k person-days.

^c^ Percent change of HCU from pre- to post-hospitalization periods due to COVID-19.

*Definition of abbreviations*: HCU: health care utilization; COVID-19: Coronavirus Disease 2019; PCP: Primary Care Provider; ICD-10-CM: International Classification for Diseases, tenth revision, clinical modification; Phys Med & Rehab: Physical Medicine and Rehabilitation; 10k = 10,000.

As a sensitivity analysis, we evaluated HCU post discharge in women and compared it to that of men patients (S5 Table in S1 File). We also analyzed HCU in patients who received care in the ICU and compared it to those who did not (S6 Table in S1 File). We found that women have higher risk of ED (rate ratio 1.03; 95% CI 1.01–1.05), rehabilitation (rate ratio 1.23; 95% CI 1.17–1.30), and telemedicine visits (rate ratio 1.12; 95% CI 1.1–1.16) than men. But women have a lower risk of inpatient visits (Rate ratio 0.91, 95% CI 0.88–0.94), and office visits (rate ratio 0.92; 95% CI 0.91–0.93) than men. Additionally, women had a shorter length of stay (rate ratio 0.84, 95% CI 0.84–0.86) (S5 Table in S1 File).

Compared to patients who did not receive ICU care, those who were admitted to the ICU have increased risk of ED visits (19%, rate ratio 1.19; 95% CI 1.17–1.22), inpatient visits (27%, rate ratio 1.27; 95% CI 1.23–1.31); shorter length of stay (34%, rate ratio 1.34; 95% CI 1.32–1.35), office visits (46%, rate ratio 1.46; 95% CI1.44–1.47), and telemedicine visits (38%, rate ratio 1.38; 95%1.35–1.43).

Contrary to the elevated risks stated above, patients discharged home from COVID-19 hospitalization who received care in the ICU were less likely to have rehabilitation visits (rate ratio 0.92; 95% CI 0.87–0.97) compared to those who did not receive care in the ICU (S6 Table in S1 File).

Outpatient office visits were the most utilized health care service by patients post discharge (275.5 visits-10k person-days). All included specialties showed more visits in the 9 months after discharge than in the 9 months prior to hospitalization. Primary care providers had the highest number of visits post discharge (133.1 visits/10k persons-days), followed by cardiology (21.9 visits/10k persons-days) and pulmonary medicine (12.9 visits/10k persons-days). Interestingly, pulmonary medicine saw the highest percent change in the number of pre- (3.8 visits/10k person-days) vs post-discharge visits (235.5% change). Similarly, the risk of having a visit to any specialty significantly increased post discharge, but the risk of having a visit with pulmonary medicine was the highest (rate ratio 3.35, 95% CI 3.26–3.45, p < .0001) (Table 3, S3, S4 Tables in S1 File).

We also found that the risk of having visits with neurology (rate ratio 1.51; 95% CI 1.46–1.58; p < .0001), psychiatry (rate ratio 1.41; 1.35–1.49; p<0.0001) and other mental health professionals (rate ratio 1.66; 95% CI 1.54–1.79; p<0.0001) significantly increased post discharge compared to the pre-hospitalization period (Table 3).

## Discussion

In our retrospective cohort study of patients discharged home after a COVID-19 hospitalization in the US, we found that these patients have an increased risk of post-discharge HCU compared to the pre-hospitalization period. Our results are similar to studies of HCU in patients post discharge from non-COVID-19 and COVID-19 admissions [3]. Our findings advance the knowledge about HCU post-COVID hospitalization in patients discharged home. Additionally, our observations of different HCU in women compared to men and in those who received care in the ICU compared to those who did not may help health systems and policy makers identify potential disparities and at-risk populations who may benefit from targeted interventions to improve their HCU post discharge. Furthermore, we found that all medical specialties studied had high use that varied by specialty, which can help us identify medical providers needed to meet patient care demands during the next respiratory pandemic.

We must consider why patients discharged home from a hospitalization for severe COVID-19 have high HCU. Acute infection by SARS-COV-2, leading to hospitalization due to severe COVID-19, has been associated with conditions that have multi-organ involvement and dysfunction, most commonly pneumonia but also including cardiac injury, acute liver injury, acute kidney injury, venous and arterial thrombotic events, and a variety of neurological and psychiatric manifestations [23]. Multisystem infection of the virus may explain a variety of persistent organ dysfunctions and may result in chronic clinical symptoms [23, 24]. These persistent symptoms likely lead patients to seek medical care post hospitalization and health systems to develop multi-disciplinary clinics, facilitating referrals to multiple specialists [25]. Our findings of increase in post-discharge HCU suggest an increased demand for hospital-centered care.

The differences in HCU in women compared to men are intriguing. Prior literature showed that women had lower risk of adverse outcomes and mortality due to COVID-19 compared to men [26, 27]. In our study, women have higher risk of ED visits, rehabilitation visits, and telemedicine visits, and lower risk of inpatient visits, office visits compared to men. Women also had a shorter length of stay compared to men. Although we do not know why these differences exist, our findings are consistent with prior non-COVID-19 literature of fewer hospital admissions, shorter length of stay, and fewer physician visits in women compared to men [28, 29]. The differences in HCU by sex may be explained by a variety of factors, including demographics and social factors, such as health care needs (limitation in mobility, disability, specific chronic comorbidities) and economic access factors (overall health, income, education, etc.) [30].

Our finding of higher HCU in patients post-discharge home from COVID-19 may be primarily driven by patients who received care in the ICU. Pre-COVID-19, research showed that patients with sepsis, pneumonia, central line associated blood stream infections, and ventilator associated pneumonia had increased post-discharge mortality and high HCU [8]. Also, patients who receive prolonged mechanical ventilation (>21 days) have high risk of mortality, readmissions to the hospital and ICU, and high HCU [5]. Finally, patients who survive ARDS have impaired functional status, and their quality of life is affected even 2 years after discharge from the ICU [7]. In our cohort, 34.5% of patients received care in the ICU, which is in accordance to published findings that nearly 1 in 3 (33%) hospitalized patients with COVID-19 develop ARDs and 1 in 4 hospitalized patients require transfer to the ICU (26%) [31]. We also know that patients with COVID-19 admitted to the ICU have longer length of stay in the hospital and ICU, longer length of mechanical ventilation, and therefore may be at increased risk of nosocomial infections [32, 33]. If we are to extrapolate those percentages to the over 6 million people hospitalized due to COVID 19, it may be expected that over 1.5 million people may experience the elevated HCU described in our study.

Notable insights emerged from our study regarding medical specialty utilization post-COVID-19 discharge. Primary care providers and outpatient office visits were pivotal, while an intriguing surge in pulmonology clinic visits highlighted disproportionate escalating demand for specialized respiratory care. This may be explained by the persistent pulmonary symptoms and complications post-COVID-19. A recent study highlighted the multisystemic impact of COVID-19 and reported that the chronic symptoms linked with SARS-CoV-2 infection involved a variety of organ systems, with chronic cough notably identified as one of the defining symptoms for the new diagnosis of post-acute sequelae of SARS-CoV-2 infection (PACS) [34]. Another study found that SARS-COV-2 infection was associated with an additional 213 health care visits per 1000 patients during the 6 months after the acute stage of illness [8]. Notably, this study found the second highest increase in utilization was observed for pulmonary symptoms (bronchitis, venous thromboembolism, dyspnea upon exertion, hypoxemia, and cough). Another study in France evaluated sequelae in COVID-19 patients post-hospital discharge from March to May 2020. They found that 51% of patients reported persistent respiratory symptoms 4 months after COVID-19 hospitalization. This study found persistent ground glass opacities in 32%, fibrotic lung lesions in 12%, and abnormal diffusion capacity in 13.6% of patients 4 months post-discharge [35]. Other studies have reported persistent reductions in diffusion capacity 3 months to 4 months after acute illness, with rates ranging from 16.4% to 52% [4, 36]. Multivariate analysis studies also found that severe disease during acute illness was associated with a persistently reduced diffusion capacity [36] and worse heart function [24]. Additionally, the most frequent serious manifestation of acute COVID-19 infection is pneumonia [37], with a reported 17% of patients complicated with acute respiratory distress syndrome [38]. All of the above may help explain our finding of 235.5% change in pulmonary visits from the pre- to the post-hospitalization period.

Interestingly, studies have reported persistent pulmonary symptoms in patients without persistent physiologic impairments [39, 40]. One study aimed to investigate the long-term pulmonary effects of severe COVID-19 pneumonia by assessing cardiopulmonary exercise test (CPET) performance in 60 patients 12 months after a COVID-19 infection that required ICU management [41]. Exercise capacity assessed by CPET was within normal limits in most patients 12 months after hospitalization, and impairment was predominantly related to persistent deconditioning or prior respiratory comorbidities. Complementing our findings, other studies have compared hospitalization related to COVID to other causes of hospitalization.

The disproportionate increases in HCU by PCP and pulmonary specialist may also be explained by the establishment of multi-disciplinary post-acute sequelae of COVID-19 (PASC) clinics. One study surveyed healthcare systems in the US participating in the PETAL Network and reported that 70% had established an outpatient clinic for PASC with physicians providing care in 97% of clinics supplemented by Advanced Practice Professionals [25]. Of these systems, 21% automatically referred all patients discharged alive post-COVID-19 hospitalization to the PASC clinic for outpatient follow up while 70% of referrals relied on physician discretion or patient requests. Subspecialties available were pulmonary (97%), general medicine and primary care (58%), cardiology (52%), and psychiatry (30%). Remarkably, 73% of these PASC clinics were distinct from their previously established post-ICU clinics [25].

We acknowledge our study’s limitations, including its retrospective design; therefore, we cannot infer cause and effect but only an association between being discharged from a hospitalization due to COVID-19 and an increase in post-discharge HCU. Also, our patient cohort was obtained from a national commercially-insured and Medicare Advantage claims database. Therefore, we do not provide information on uninsured/out of network patients, and this may underrepresent the number of patients discharged home from COVID-19 hospitalizations and their post-discharge HCU. Also, our study period spans the first year of the pandemic when many COVID-19 treatments and vaccines were in development or in early stages of use. Therefore, we are unable to determine how many of our patients were vaccinated or had received COVID-19-specific therapy and how these two factors mediate post-discharge HCU. Also, the mean age of our population is 72 years, and our results may be more representative of the older adult population. Similarly, based on limitations from our dataset, we cannot determine the post-discharge mortality rate in our cohort; however, with an estimated 7.8% all-cause post-discharge mortality rate [42] reported in the literature, we know most patients discharged from a COVID-19 hospitalization are alive one year post-discharge [42]. To address this limitation, our cohort only included patients enrolled in insurance/database until the end of the study period. Therefore, each patient contributed person-days until death, loss of insurance eligibility, or the end of the 9-month follow-up period. Additionally, we acknowledge the multifaceted influences on healthcare utilization, extending beyond direct medical necessity. Integral factors likely include socioeconomic status, health insurance coverage, access to and availability of care, and clinician referrals. We must also consider the potential for bias due to resource exhaustion amid the pandemic’s economic fallout. Resource limitation might have prompted a decrease in healthcare visits despite ongoing health impairments, thereby affecting our analysis of utilization rates.

Our study has several strengths including a cohort of patients discharged home obtained from a database that spans nationally. The information obtained from our study provides insights in the HCU trends post-COVID-19 hospitalization in the US. We were also able to follow patients for a considerable period, up to 9 months post discharge, and to compare pre- and post-COVID hospitalization HCU in the same patient population, eliminating potential selection bias or the influence of such confounders as demographic characteristics and prior comorbidities.

## Conclusions and implications

In our nationally representative retrospective study, we identified that HCU remains high among patients discharged to a home setting after a hospitalization due to COVID-19. Health systems and providers may be able to use this information to better deploy resources in the care of this chronically ill population.

## Supporting information

S1 File This document contains supplementary tables and figures that provide additional details and analyses supporting the findings of the main manuscript. The tables and figures included herein offer further insights, data points, and visual representations to enhance the understanding and interpretation of the research presented in the primary manuscript.
(DOCX)

## References

[1] Centers of Disease Control and Prevention COVID Data Tracker: Hospital Admissions [Internet]. [cited 2022 Sep 11]. Available from: https://covid.cdc.gov/covid-data-tracker/#new-hospital-admissions

[2] Lavery AM, Preston LE, Ko JY, Chevinsky JR, DeSisto CL, Pennington AF, et al. Characteristics of Hospitalized COVID-19 Patients Discharged and Experiencing Same-Hospital Readmission—United States, March-August 2020. MMWR Morb Mortal Wkly Rep [Internet]. 2020 Nov 13 [cited 2023 Jan 20];69(45):1695–9. Available from: https://pubmed.ncbi.nlm.nih.gov/33180754/ doi: 10.15585/mmwr.mm6945e2 33180754 PMC7660660

[3] Koumpias AM, Schwartzman D, Fleming O. Long-haul COVID: healthcare utilization and medical expenditures 6 months post-diagnosis. BMC Health Serv Res [Internet]. 2022 Dec 1 [cited 2023 Jun 25];22(1):1–14. Available from: https://bmchealthservres.biomedcentral.com/articles/10.1186/s12913-022-08387-3 35941617 10.1186/s12913-022-08387-3 PMC9358916

[4] miao Zhao Y, min Shang Y, bin Song W, quan Li Q, Xie H, fu Xu Q, et al. Follow-up study of the pulmonary function and related physiological characteristics of COVID-19 survivors three months after recovery. EClinicalMedicine [Internet]. 2020 Aug 1 [cited 2022 Sep 11];25:100463. Available from: /pmc/articles/PMC7361108/ doi: 10.1016/j.eclinm.2020.100463 32838236 PMC7361108

[5] Hill AD, Fowler RA, Burns KEA, Rose L, Pinto RL, Scales DC. Long-Term Outcomes and Health Care Utilization after Prolonged Mechanical Ventilation. Ann Am Thorac Soc [Internet]. 2017 Mar 1 [cited 2023 Aug 16];14(3):355–62. Available from: https://pubmed.ncbi.nlm.nih.gov/28033033/ doi: 10.1513/AnnalsATS.201610-792OC 28033033

[6] Hill AD, Fowler RA, Pinto R, Herridge MS, Cuthbertson BH, Scales DC. Long-term outcomes and healthcare utilization following critical illness—a population-based study. Crit Care [Internet]. 2016 Mar 31 [cited 2022 Sep 11];20(1). Available from: https://pubmed.ncbi.nlm.nih.gov/27037030/ doi: 10.1186/s13054-016-1248-y 27037030 PMC4818427

[7] Cheung AM, Tansey CM, Tomlinson G, Diaz-Granados N, Matté A, Barr A, et al. Two-year outcomes, health care use, and costs of survivors of acute respiratory distress syndrome. Am J Respir Crit Care Med [Internet]. 2006 Sep 1 [cited 2023 Aug 16];174(5):538–44. Available from: https://pubmed.ncbi.nlm.nih.gov/16763220/ doi: 10.1164/rccm.200505-693OC 16763220

[8] Dick A, Liu H, Zwanziger J, Perencevich E, Furuya EY, Larson E, et al. Long-term survival and healthcare utilization outcomes attributable to sepsis and pneumonia. BMC Health Serv Res [Internet]. 2012 [cited 2023 Aug 16];12(1):432. Available from: /pmc/articles/PMC3534544/10.1186/1472-6963-12-432 PMC3534544 23181764

[9] Tartof SY, Malden DE, Liu ILA, Sy LS, Lewin BJ, Williams JTB, et al. Health Care Utilization in the 6 Months Following SARS-CoV-2 Infection. JAMA Netw Open [Internet]. 2022 Aug 1 [cited 2023 Jan 20];5(8):e2225657–e2225657. Available from: https://jamanetwork.com/journals/jamanetworkopen/fullarticle/2795163 doi: 10.1001/jamanetworkopen.2022.25657 35960522 PMC9375168

[10] McNaughton CD, Austin PC, Sivaswamy A, Fang J, Abdel-Qadir H, Daneman N, et al. Post-acute health care burden after SARS-CoV-2 infection: a retrospective cohort study. CMAJ [Internet]. 2022 Oct 17 [cited 2023 Jan 20];194(40):E1368–76. Available from: https://pubmed.ncbi.nlm.nih.gov/36252983/10.1503/cmaj.220728 PMC9616149 36252983

[11] McAlister FA, Dong Y, Chu A, Wang X, Youngson E, Quinn KL, et al. The risk of death or unplanned readmission after discharge from a COVID-19 hospitalization in Alberta and Ontario. CMAJ [Internet]. 2022 May 16 [cited 2023 Jan 20];194(19):E666–73. Available from: https://pubmed.ncbi.nlm.nih.gov/35577377/ doi: 10.1503/cmaj.220272 35577377 PMC9438727

[12] Loo WK, Hasikin K, Suhaimi A, Yee PL, Teo K, Xia K, et al. Systematic Review on COVID-19 Readmission and Risk Factors: Future of Machine Learning in COVID-19 Readmission Studies. Front Public Health. 2022 May 23;10:1311. doi: 10.3389/fpubh.2022.898254 35677770 PMC9168237

[13] Robinson-Lane SG, Sutton NR, Chubb H, Yeow RY, Mazzara N, DeMarco K, et al. Race, Ethnicity, and 60-Day Outcomes After Hospitalization With COVID-19. J Am Med Dir Assoc [Internet]. 2021 Nov 1 [cited 2023 Jan 20];22(11):2245–50. Available from: https://pubmed.ncbi.nlm.nih.gov/34716006/ doi: 10.1016/j.jamda.2021.08.023 34716006 PMC8490827

[14] Gwin M, Saleki M, Lampert H, Meo N, Bann M. Emergency department visits and readmissions after COVID-19 hospitalization: a cross-sectional analysis. Intern Emerg Med [Internet]. 2021 Sep 1 [cited 2023 Jan 20];16(6):1715–8. Available from: https://pubmed.ncbi.nlm.nih.gov/33537919/ doi: 10.1007/s11739-021-02644-9 33537919 PMC7857343

[15] Taupin D, Anderson TS, Merchant EA, Kapoor A, Sokol-Hessner L, Yang JJ, et al. Preventability of 30-Day Hospital Revisits Following Admission with COVID-19 at an Academic Medical Center. Jt Comm J Qual Patient Saf [Internet]. 2021 Nov 1 [cited 2023 Jan 20];47(11):696–703. Available from: https://pubmed.ncbi.nlm.nih.gov/34548237/ doi: 10.1016/j.jcjq.2021.08.011 34548237 PMC8383478

[16] Donnelly JP, Wang XQ, Iwashyna TJ, Prescott HC. Readmission and Death After Initial Hospital Discharge Among Patients With COVID-19 in a Large Multihospital System. JAMA [Internet]. 2021 Jan 19 [cited 2023 Jan 20];325(3):304–6. Available from: https://jamanetwork.com/journals/jama/fullarticle/2774380 doi: 10.1001/jama.2020.21465 33315057 PMC7737131

[17] Atalla E, Kalligeros M, Giampaolo G, Mylona EK, Shehadeh F, Mylonakis E. Readmissions among patients with COVID-19. Int J Clin Pract [Internet]. 2021 Mar 1 [cited 2023 Jan 20];75(3):e13700. Available from: https://onlinelibrary.wiley.com/doi/full/10.1111/ijcp.13700 32894801 10.1111/ijcp.13700

[18] Zhang Y, Khullar D, Wang F, Steel P, Wu Y, Orlander D, et al. Socioeconomic variation in characteristics, outcomes, and healthcare utilization of COVID-19 patients in New York City. PLoS One. 2021 Jul 1;16(7):e0255171. doi: 10.1371/journal.pone.0255171 34324574 PMC8321227

[19] Roth SE, Govier DJ, Marsi K, Cohen-Cline H. Differences in Outpatient Health Care Utilization 12 Months after COVID-19 Infection by Race/Ethnicity and Community Social Vulnerability. Int J Environ Res Public Health [Internet]. 2022 Mar 1 [cited 2023 Aug 6];19(6). Available from: /pmc/articles/PMC8949439/ doi: 10.3390/ijerph19063481 35329165 PMC8949439

[20] Chopra V, Flanders SA, O’Malley M, Malani AN, Prescott HC. Sixty-Day Outcomes Among Patients Hospitalized With COVID-19. Ann Intern Med [Internet]. 2021 Apr 1 [cited 2023 Jan 31];174(4):576–8. Available from: /pmc/articles/PMC7707210/ doi: 10.7326/M20-5661 33175566 PMC7707210

[21] Zaidan MF, Neira DAP, Polychronopoulou E, Nishi SP, Duarte AG, Kuo YF, et al. Health care utilization among patients discharged alive post-COVIDF-19 hospitalization. Chest [Internet]. 2022 Oct [cited 2023 Aug 21];162(4):A519. Available from: /pmc/articles/PMC9548925/

[22] Hardeo Sahai, Anwer Khurshid. Statistics in epidemiology: methods, techniques, and applications. 1996;321.

[23] Wiersinga WJ, Rhodes A, Cheng AC, Peacock SJ, Prescott HC. Pathophysiology, Transmission, Diagnosis, and Treatment of Coronavirus Disease 2019 (COVID-19): A Review. JAMA [Internet]. 2020 Aug 25 [cited 2022 Sep 11];324(8):782–93. Available from: https://jamanetwork.com/journals/jama/fullarticle/2768391 doi: 10.1001/jama.2020.12839 32648899

[24] Berlin DA, Gulick RM, Martinez FJ. Severe Covid-19. New England Journal of Medicine [Internet]. 2020 Dec 17 [cited 2022 Sep 11];383(25):2451–60. Available from: https://www.nejm.org/doi/full/ doi: 10.1056/NEJMcp2009575 32412710

[25] Valley TS, Schutz A, Peltan ID, Vranas KC, Mathews KS, Jolley SE, et al. Organization of Outpatient Care After COVID-19 Hospitalization. Chest [Internet]. 2022 Jan 31 [cited 2022 Sep 11];161(6):1485–9. Available from: https://europepmc.org/articles/PMC8801392 doi: 10.1016/j.chest.2022.01.034 35108571 PMC8801392

[26] Jin JM, Bai P, He W, Wu F, Liu XF, Han DM, et al. Gender Differences in Patients With COVID-19: Focus on Severity and Mortality. Front Public Health. 2020 Apr 29;8:545030. doi: 10.3389/fpubh.2020.00152 32411652 PMC7201103

[27] Grasselli G, Greco M, Zanella A, Albano G, Antonelli M, Bellani G, et al. Risk Factors Associated With Mortality Among Patients With COVID-19 in Intensive Care Units in Lombardy, Italy. JAMA Intern Med [Internet]. 2020 Oct 1 [cited 2023 Aug 19];180(10):1345–55. Available from: https://pubmed.ncbi.nlm.nih.gov/32667669/ doi: 10.1001/jamainternmed.2020.3539 32667669 PMC7364371

[28] Cameron KA, Song J, Manheim LM, Dunlop DD. Gender Disparities in Health and Healthcare Use Among Older Adults. J Womens Health [Internet]. 2010 Sep 1 [cited 2023 Aug 17];19(9):1643. Available from: /pmc/articles/PMC2965695/ doi: 10.1089/jwh.2009.1701 20695815 PMC2965695

[29] Lum YS, Chang HJ. The effect of Medicaid coverage on use of health services by low-income elderly people. Soc Work Res [Internet]. 1998 Mar 1 [cited 2023 Aug 19];22(1):31–43. Available from: doi: 10.1093/swr/22.1.31

[30] Danielsen AC, Lee KM, Boulicault M, Rushovich T, Gompers A, Tarrant A, et al. Sex disparities in COVID-19 outcomes in the United States: Quantifying and contextualizing variation. Soc Sci Med. 2022 Feb 1;294:114716. doi: 10.1016/j.socscimed.2022.114716 35042136 PMC8743486

[31] Tzotzos SJ, Fischer B, Fischer H, Zeitlinger M. Incidence of ARDS and outcomes in hospitalized patients with COVID-19: a global literature survey. Crit Care [Internet]. 2020 Aug 21 [cited 2023 Aug 16];24(1). Available from: /pmc/articles/PMC7441837/ doi: 10.1186/s13054-020-03240-7 32825837 PMC7441837

[32] Oliveira E, Parikh A, Lopez-Ruiz A, Carrilo M, Goldberg J, Cearras M, et al. ICU outcomes and survival in patients with severe COVID-19 in the largest health care system in central Florida. PLoS One [Internet]. 2021 Mar 1 [cited 2023 Aug 16];16(3):e0249038. Available from: https://journals.plos.org/plosone/article?id=10.1371/journal.pone.0249038 33765049 10.1371/journal.pone.0249038 PMC7993561

[33] Gendreau S, Benelli B, Delíere M, Tuffet S, de Prost N, Razazi K, et al. Partitioning Mechanical Ventilator Duration in COVID-19-related Acute Respiratory Distress Syndrome. Am J Respir Crit Care Med. 2022 Jul 1;206(1):114–8. doi: 10.1164/rccm.202108-1963LE 35394404 PMC9954339

[34] Thaweethai T, Jolley SE, Karlson EW, Levitan EB, Levy B, McComsey GA, et al. Development of a Definition of Postacute Sequelae of SARS-CoV-2 Infection. JAMA [Internet]. 2023 Jun 13 [cited 2023 Aug 16];329(22):1934–46. Available from: https://jamanetwork.com/journals/jama/fullarticle/2805540 doi: 10.1001/jama.2023.8823 37278994 PMC10214179

[35] Morin L, Savale L, Pham T, Colle R, Figueiredo S, Harrois A, et al. Four-Month Clinical Status of a Cohort of Patients After Hospitalization for COVID-19. JAMA [Internet]. 2021 Apr 20 [cited 2022 Sep 11];325(15):1525–34. Available from: https://pubmed.ncbi.nlm.nih.gov/33729425/ doi: 10.1001/jama.2021.3331 33729425 PMC7970386

[36] Blanco JR, Cobos-Ceballos MJ, Navarro F, Sanjoaquin I, Arnaiz de las Revillas F, Bernal E, et al. Pulmonary long-term consequences of COVID-19 infections after hospital discharge. Clinical Microbiology and Infection [Internet]. 2021 Jun 1 [cited 2022 Sep 11];27(6):892. Available from: /pmc/articles/PMC7920814/ doi: 10.1016/j.cmi.2021.02.019 33662544 PMC7920814

[37] Zaim S, Chong JH, Sankaranarayanan V, Harky A. COVID-19 and Multiorgan Response. Curr Probl Cardiol [Internet]. 2020 Aug 1 [cited 2022 Sep 9];45(8):100618. Available from: /pmc/articles/PMC7187881/ doi: 10.1016/j.cpcardiol.2020.100618 32439197 PMC7187881

[38] Chen J, Qi T, Liu L, Ling Y, Qian Z, Li T, et al. Clinical progression of patients with COVID-19 in Shanghai, China. J Infect [Internet]. 2020 May 1 [cited 2022 Sep 11];80(5):e1–6. Available from: https://pubmed.ncbi.nlm.nih.gov/32171869/ doi: 10.1016/j.jinf.2020.03.004 32171869 PMC7102530

[39] Gamberini L, Mazzoli CA, Prediletto I, Sintonen H, Scaramuzzo G, Allegri D, et al. Health-related quality of life profiles, trajectories, persistent symptoms and pulmonary function one year after ICU discharge in invasively ventilated COVID-19 patients, a prospective follow-up study. Respir Med [Internet]. 2021 Nov 1 [cited 2023 Jan 21];189. Available from: https://pubmed.ncbi.nlm.nih.gov/34717097/ doi: 10.1016/j.rmed.2021.106665 34717097 PMC8531241

[40] Scaramuzzo G, Ronzoni L, Campo G, Priani P, Arena C, La Rosa R, et al. Long-term dyspnea, regional ventilation distribution and peripheral lung function in COVID-19 survivors: a 1 year follow up study. BMC Pulm Med [Internet]. 2022 Nov 9 [cited 2023 Jan 21];22(1). Available from: https://pubmed.ncbi.nlm.nih.gov/36352423/10.1186/s12890-022-02214-5 PMC9643983 36352423

[41] Noureddine S, Roux-Claudé P, Laurent L, Ritter O, Dolla P, Karaer S, et al. Evaluation of long-term sequelae by cardiopulmonary exercise testing 12 months after hospitalization for severe COVID-19. BMC Pulm Med [Internet]. 2023 Dec 1 [cited 2023 Jan 21];23(1). Available from: https://pubmed.ncbi.nlm.nih.gov/36635717/10.1186/s12890-023-02313-x PMC9834678 36635717

[42] Ramzi ZS. Hospital readmissions and post-discharge all-cause mortality in COVID-19 recovered patients; A systematic review and meta-analysis. Am J Emerg Med [Internet]. 2022 Jan 1 [cited 2023 Jan 23];51:267–79. Available from: https://pubmed.ncbi.nlm.nih.gov/34781153/ doi: 10.1016/j.ajem.2021.10.059 34781153 PMC8570797

