# Supplementary material for: Healthcare utilization 9 months pre- and post- COVID-19 hospitalization among patients discharged alive
Source: PLoS One. 2024 Jun 20;19(6):e0303509. doi: 10.1371/journal.pone.0303509 (PMC11189225; doi:10.1371/journal.pone.0303509)
Supplement: S1 File — The tables and figures included herein offer further insights, data points, and visual representations to enhance the understanding and interpretation of the research presented in the primary manuscript. (DOCX) [file pone.0303509.s001.docx]

# **Supporting Information**

**S1 Table. Health care utilization of patients discharged alive, divided in 3-month periods pre and post-hospitalization due to COVID-19.** 

|  | **Pre-COVID-19 Hospitalization ^a,b^** | | | **Post-COVID-19 Hospitalization ^a,b^** | | |
| --- | --- | --- | --- | --- | --- | --- |
|  | -6-9 months | -3-6 months | -0-3 months | +0-3 months | +3-6 months | +6-9 months |
| **ED** | 10.7 | 18.2 | 37.7 | 43.0 | 24.4 | 20.9 |
| **Inpatient admission** | 4.3 | 7.4 | 9.4 | 22.3 | 10.1 | 8.2 |
| **Inpatient admission (los)** | 24.9 | 47.0 | 57.1 | 169.5 | 66.5 | 54.3 |
| **Rehab/SNF** | 1.9 | 3.4 | 4.8 | 7.8 | 3.8 | 3.2 |
| **Outpatient office** | 102.7 | 169.8 | 199.9 | 294.5 | 239.8 | 197.2 |
| **Telemedicine** | 9.4 | 10.1 | 14.2 | 26.8 | 9.2 | 7.5 |

^a^ Health care utilization of patients, measured by number of visits per 10k person-days.

^b^ All adults discharged home from a hospitalization due to primary diagnosis of COVID-19 between April 2020 and March 2021, with at least 12 months continuous enrollment before COVID-19 were included.

*Definition of abbreviations:* COVID-19: Coronavirus Disease 2019; ED: Emergency Department; ICD-10-CM: International Classification for Diseases, tenth revision, clinical modification; LOS: length of stay; Rehab/snf: rehabilitation/ skilled nursing facility; 10k=10,000

**S2 Table.** **Health care utilization of patients pre and post hospitalization due to COVID-19, by number of unique patients.** 

|  | **Pre-COVID-19 Hospitalization ^a,b^** | **Post-Covid-19 Hospitalization ^a,b^** | | |
| --- | --- | --- | --- | --- |
|  | -0-9 months | +0-3 months | +3-+6 months | +6-9 months |
| **ED visits** | 21,247 | 15,201 | 7,184 | 2,974 |
| **Inpatient visits** | 8,161 | 9,280 | 3,432 | 1,331 |
| **Rehab/snf visits** | 4,028 | 3,458 | 1,389 | 543 |
| **Outpatient visits** | 48,365 | 45,825 | 35,582 | 15,910 |
| **Telemedicine visits** | 11,183 | 9,009 | 2,796 | 1,083 |

^a^ Our cohort consists of 63,161 patients discharged home after hospitalization due to COVID-19.

^b^ Health care utilization of patients, measured by number of visits per 10k person-days.

*Definition of abbreviations:* COVID-19: Coronavirus Disease 2019; ED: Emergency Department; ICD-10-CM: International Classification for Diseases, tenth revision, clinical modification; Rehab/snf: rehabilitation/ skilled nursing facility; 10k=10,000

**S3 Table.** **Health Care Utilization of Patients Pre And Post-Hospitalization due to COVID-19 by medical specialty.** 

| **-** | **Pre-COVID-19 Hospitalization ^a,b^** | | | **Post-Covid-19 Hospitalization ^a,b^** | | |
| --- | --- | --- | --- | --- | --- | --- |
| **Medical Specialty** | **-6-9 months** | **-3-6 months** | **-0-3 months** | **+0-3 months** | **+3-6 months** | **+6-9 months** |
| **PCP** | 106.7 | 79.0 | 52.3 | 176.7 | 151.0 | 144.4 |
| **Cardiology** | 13.9 | 13.9 | 9.0 | 25.7 | 29.3 | 26.5 |
| **Pulmonary Medicine** | 4.6 | 4.2 | 2.7 | 17.2 | 15.4 | 11.9 |
| **Endocrinology** | 2.5 | 2.5 | 1.7 | 4.0 | 5.1 | 5.2 |
| **Neurology** | 3.3 | 3.4 | 2.3 | 4.7 | 6.7 | 6.5 |
| **Phys Med & Rehab** | 2.2 | 2.3 | 1.5 | 2.4 | 3.7 | 4.0 |
| **Psychiatry** | 2.2 | 2.1 | 1.6 | 2.9 | 3.7 | 4.3 |
| **Mental Health Professional** | 0.9 | 0.9 | 0.6 | 1.5 | 1.8 | 1.9 |

^a^ HCU measured by number of office visits by specialty per 10k person-days

^b^ All adults discharged home from a hospitalization due to primary diagnosis of COVID-19 between April 2020 and March 2021, with at least 12 months continuous enrollment before COVID-19 were included.

*Definition of abbreviations:* HCU: health care utilization; COVID-19: Coronavirus Disease 2019; LOS: Length of stay; ED: Emergency department ICD-10-CM: International Classification for Diseases, tenth revision, clinical modification; 10k=10,000; PCP: primary care provider; Phys Med & Rehab: Physical medicine and rehabilitation.

**S4 Table.** **Health care utilization of patients discharged alive, divided in 3-month periods before and after hospitalization due to COVID-19, by medical specialty and number of unique patients.**  

|  | **Pre-COVID-19 ^a,b^** | | | **Post-COVID-19 ^a,b^** | | |
| --- | --- | --- | --- | --- | --- | --- |
| **Medical Specialty** | **-6-9 months** | **-3-6 months** | **-0-3 months** | **+0-3 months** | **+3-6 months** | **+6-9 months** |
| **PCP** | 60,630 | 44,898 | 31,407 | 88,928 | 46,362 | 19,254 |
| **Cardiology** | 7,889 | 7,920 | 5,426 | 12,911 | 8,988 | 3,539 |
| **Pulmonary Medicine** | 2,633 | 2,405 | 1,646 | 8,654 | 4,740 | 1,593 |
| **Endocrinology** | 1,429 | 1,446 | 1,049 | 2,036 | 1,563 | 698 |
| **Neurology** | 1,886 | 1,952 | 1,355 | 2,345 | 2,048 | 870 |
| **Phys Med & Rehab** | 1,275 | 1,294 | 879 | 1,220 | 1,146 | 534 |
| **Psychiatry** | 1,230 | 1,188 | 941 | 1,457 | 1,148 | 570 |
| **Mental Health Professional** | 530 | 518 | 363 | 747 | 563 | 256 |

^a^ Our cohort consists of 63,161 patients discharged home after hospitalization due to COVID-19 between April 2020 and March 2021, with at least 12 months of continuous enrollment before COVID-19.

^b^ We evaluated HCU in the 9 months pre and post hospitalization from COVID-19.

*Definition of abbreviations:* HCU: health care utilization; COVID-19: Coronavirus Disease 2019; LOS: Length of stay; ED: Emergency department ICD-10-CM: International Classification for Diseases, tenth revision, clinical modification; 10k=10,000; PCP: primary care provider; Phys Med & Rehab: Physical medicine and rehabilitation.

**S5 Table.** **Health care utilization of patients pre and post hospitalization due to COVID-19 by sex.** 

| **-** | **HCU Men** | | **-** | **HCU Women** | | **-** | **-** |
| --- | --- | --- | --- | --- | --- | --- | --- |
| **-** | **Pre-COVID-19** | **Post- COVID-19** | **-** | **Pre-COVID-19** | **Post- COVID-19** | **-** | **-** |
| **-** | -0-9 months | +0-9 months | Percent change | -0-9 months | +0-9 months | Percent change | Rate Ratio Post-Covid Males Vs Females |
| **ED Visits** | 21.33 | 31.90 | 49.53 | 22.89 | 32.96 | 43.98 | 0.97 (0.95-0.99) |
| **Inpatient Visits** | 6.88 | 16.15 | 134.74 | 7.16 | 14.72 | 105.44 | 1.10 (1.07-1.13) |
| **Inpatient Admission (Los)** | 42.30 | 121.42 | 187.05 | 43.29 | 103.35 | 138.72 | 1.17 (1.16-1.19) |
| **Rehab/SNF Visits** | 2.54 | 4.68 | 83.91 | 3.52 | 5.78 | 64.09 | 0.81 (0.77-0.85) |
| **Office Visits** | 158.75 | 266.01 | 67.57 | 159.19 | 252.46 | 58.59 | 1.05 (1.04-1.06) |
| **Telemedicine Visits** | 9.99 | 15.94 | 59.47 | 12.51 | 17.90 | 43.07 | 0.89 (0.87-0.92) |

*Definition of abbreviations:* HCU: health care utilization; COVID-19: Coronavirus Disease 2019; LOS: Length of stay; ED: Emergency department ICD-10-CM: International Classification for Diseases, tenth revision, clinical modification; Rehab/snf: Rehabilitation/Skilled nursing facility; 10k=10,000.

**S6 Table.** **Health Care Utilization of Patients Pre And Post-Hospitalization due to COVID-19 by ICU.** 

| - | **No ICU** | | **-** | **ICU** | | **-** | **-** |
| --- | --- | --- | --- | --- | --- | --- | --- |
| **-** | **Pre-COVID-19** | **Post COVID-19** | **-** | **Pre- COVID-19** | **Post COVID-19** | **-** | **-** |
| **-** | -0-9 months | +0-9 months | Percent change | -0-9 months | +0-9 months | Percent change | Rate ratio post COVID-19 ICU vs non-ICU |
| **ED visits** | 22.54 | 32.11 | 42.48 | 21.48 | 38.31 | 78.34 | 1.19 (1.17-1.22) |
| **Inpatient visits** | 7.14 | 14.86 | 108.11 | 6.84 | 18.91 | 176.64 | 1.27 (1.23-1.31) |
| **Inpatient admission (los)** | 44.31 | 105.86 | 138.90 | 40.04 | 141.71 | 253.91 | 1.34 (1.32-1.35) |
| **Rehab/SNF visits** | 3.61 | 5.68 | 57.18 | 2.04 | 5.21 | 155.86 | 0.92 (0.87-0.97) |
| **Office visits** | 152.36 | 237.37 | 55.79 | 171.64 | 345.58 | 101.34 | 1.46 (1.44-1.47) |
| **Telemedicine visits** | 10.95 | 15.91 | 45.32 | 12.11 | 22.03 | 81.90 | 1.38 (1.35-1.43) |

*Definition of abbreviations:* HCU: health care utilization; COVID-19: Coronavirus Disease 2019; LOS: Length of stay; ED: Emergency department ICD-10-CM: International Classification for Diseases, tenth revision, clinical modification; Rehab/snf: Rehabilitation/Skilled nursing facility; 10k=10,000.

**S7 Table. Disease Comorbidities and ICD-10 codes**

| **Disease** | **ICD-10-CM codes** |
| --- | --- |
| COVID-19 | U07.1 |
| Diabetes | E10.xx, E11.xx, E13.xx |
| Hypertension | I10.x, I11.xx, I12.xx, I13.xx I15.xx, I67.4 |
| Asthma | J45.xx |
| COPD | J41.8, J42.xx, J43.xx, J44.xx |
| CKD | N18.9 |
| ESRD | N18.6 |
| Stroke | I63.xx, I64.xx, I69.3x, G45.9 |
| Heart disease | I09.9, I11.0, I13.xx, I25.5, I42.xx, I43.xx, I50.xx |
| Cancer | C00.x – C96.x |
| CAD | I25.1X |
| Liver disease | K70 – K74, K76.xx |

*Definition of abbreviations:* ICD-10-CM: International Classification for Diseases, tenth revision, clinical modification ; COVID-19: Coronavirus Disease 2019; COPD: Chronic obstructive pulmonary disease; CKD: Chronic kidney disease. ESRD: End-stage redal disease; CAD: Coronary artery disease.

**S8 Table. Health Care Utilization of Patients Pre And Post-Hospitalization due to COVID-19 by age**

| **Age** | **<65** | | | **>=65** | | | **Rate ratio post-covid >=65 vs<65** |
| --- | --- | --- | --- | --- | --- | --- | --- |
|  | **Pre-covid** | **Post covid** | **Percent change** | **Pre-covid** | **Post covid** | **Percent change** |  |
|  | **-0-9 months** | **+0-9 months** |  | **-0-9 months** | **+0-9 months** |  |  |
| **Inpatient admission (los)** | 33.95 | 139.66 | 311.32 | 45.31 | 173.94 | 283.86 | 1.25 (1.23-1.26) |
| **Inpatient visits** | 5.67 | 19.24 | 239.40 | 7.41 | 23.95 | 223.07 | 1.24 (1.20-1.29) |
| **ED visits** | 25.33 | 49.81 | 96.69 | 21.30 | 48.16 | 126.12 | 0.97 (0.94-0.99) |
| **Rehab visits** | 1.10 | 2.94 | 167.46 | 3.62 | 9.18 | 153.79 | 3.12 (2.85-3.44) |
| **Office visits** | 155.87 | 444.66 | 185.28 | 159.87 | 370.97 | 132.04 | 0.83 (0.82-.84) |
| **Telemedicine visits** | 8.67 | 22.45 | 158.87 | 12.09 | 26.17 | 116.39 | 1.17 (1.12-1.21) |

*Definition of abbreviations*: COVID-19: Coronavirus Disease 2019; LOS: Length of stay; ED: Emergency department
