# Supplementary material for: Healthcare utilization 9 months pre- and post- COVID-19 hospitalization among patients discharged alive
Source: PLoS One. 2024 Jun 20;19(6):e0303509. doi: 10.1371/journal.pone.0303509 (PMC11189225; doi:10.1371/journal.pone.0303509)
Supplement: Attachment — Submitted filename: Response to Reviewers.docx [file pone.0303509.s002.docx]

**Response to Reviewers**

Please use the space provided to explain your answers to the questions above. You may also include additional comments for the author, including concerns about dual publication, research ethics, or publication ethics. (Please upload your review as an attachment if it exceeds 20,000 characters)

**Overlapping text:**

“We noticed you have some minor occurrence of overlapping text with the following previous publication(s), which needs to be addressed:

<https://www.ncbi.nlm.nih.gov/pmc/articles/PMC9548925/>

<https://bmcpulmmed.biomedcentral.com/articles/10.1186/s12890-023-02313-x>

<https://www.jamda.com/article/S1525-8610(21)00762-3/fulltext>

**Response**:

Thank you for bringing this to our attention.

The first link is our submitted abstract to the CHEST conference in 2022. We have added a reference to this abstract.
We have also edited the manuscript to avoid any further overlapping texts.

**Reviewer Comments**

**Reviewer #1:**

**C1:** “The authors use a large health system in the US to describe patterns in health care use among individuals who were hospitalized with COVID-19 in the early phases of the pandemic, prior to widespread vaccine access or the newer variants. Although they describe exposure and outcome, formal comparisons are not included. It would be of potential interest if, for example, they compared post-hospitalization use to other conditions (e.g., sepsis, influenza), pre- vs. post-HCU, or even formally assessed trends in HCU over time. At times the authors refer to people who were hospitalized but not discharged home as being excluded, but most of Table 2 describes alternative dispositions for patients who were not discharged home. Perhaps another formal comparison would be of interest in that regard, e.g., predictors of home vs. not-home discharge.”

**R1:** Thank you for your comment. Our study is based on a cohort of patients discharged home from a hospitalization due to COVID-19. Our table 2 describes the discharge destination of all patients post hospitalization, including our cohort of patients discharged home. Our study focuses on patients who have been discharged home and not the patients discharged to other destinations. Therefore, we have decided to delete table 2.

We have performed formal comparisons of health care utilization (HCU) pre and post- COVID-19 -hospitalization and now describe rate ratios and p values. See table 2 and 3 of our main manuscript and S5-S6 tables in the supplement material.

We considered the suggestion of comparing to other potentially similar conditions. This was a great question to investigate, however, we felt this would not be fully in line with this study

We also considered the suggestion of evaluating for predictors of home vs non-home discharge, and the possibility of comparing post-COVID-19 hospitalization use with that of other conditions. We also thought this would not be fully in line with this study.

**C2:** “It is important to note in more detail that health care use in the US is not driven solely by patient need for medical care. It involves a complex interplay of multiple factors, including health insurance, access to care, available medical care, and referrals. Patients cannot access medical care they (or their clinicians) do not know they would benefit from (e.g., increasing evidence for increased risk of diabetes following infection suggests some patients might benefit from endocrinology evaluation; GI complications are also apparent, as are fertility issues, but few were aware of these issues at the time of the study); second, patients who have exhausted available resources do eventually stop going to as many medical appointments, even in cases where their health remains severely impaired. Please comment.

**R2:** Thank you for your comment. We agree with the reviewer, the multifaceted influences on healthcare utilization extend beyond direct medical necessity. We have added statements to reflect the above-mentioned interplay of multiple factors in the study limitations. Please see the limitations section under our discussion.

**C3:** “With regards to healthcare encounters, is it possible to include home health encounters?
 Did the authors consider looking for potential effect modification by sex? Or by ICU admission during the index hospitalization? What % of cases were re-infections, and if an important %, what if any difference is there in HCU by reinfection?”

**R3:** Thank you for your comment. Unfortunately, we are unable to provide information regarding home health encounters or data on re-infections as these are difficult to discern on claims databases. However, as a sensitivity analysis, we now evaluate the HCU by gender and ICU admission. Please see S5-S6 Table in the supplement material.

**C4**: “The authors describe covariates, although no adjusted models were run. Perhaps baseline patient characteristics might be a more accurate term. With regards to these variables, why gender (instead of sex), what were the race categories (and were they patient reported or something else), and what about other important potential confounders, e.g., education, insurance, occupation, number of vaccinations? Finally, inclusion of comorbid conditions only in the prior 12 months likely significantly underestimated true co-morbid conditions, is it possible to expand the look-back window to several years?

Table 1: What % were covered by commercial vs. other health insurance?

Table 2 seems more appropriate for study flow- these patients were excluded from the cohort.

**R4:** Thank you for your comments. We agree the term “baseline characteristics” is a more accurate term and this has been changed. Please see table 1 in our main manuscript.

Gender has been switched to sex throughout the manuscript.

We obtained the information of race/ethnicity as was noted by their insurance. Patients self-identifying as non-Hispanic ethnicity were categorized based on race (White, Black, Asian, other/unknown). Patients self-identifying as Hispanic ethnicity were included in the Hispanic group regardless of race. Please see Table 1 footnote in our main manuscript document.

We agree regarding other potential cofounders (education, insurance, occupation, number of vaccinations) in the evaluation of HCU post-discharge from COVID-19 or any hospitalization. We do not have access to these multiple socioeconomic variables. We have added this to the limitations section in our discussion.

We do not have the specific information for our cohort regarding the percentage of patients covered by commercial vs. other health insurance. However, we do know however that 10% of Optum enrollees have Medicare Advantage.

We have deleted table 2 as it did not add any important information for our current study design.

We are unable to expand the time frame of comorbidities prior to 12 months from index hospitalization as our contract to access CDM raw data ended on 6/30/2023.

**C5:** There are more limitations than the authors list, including the following:
 - Use of only patients insured by a large commercial company or Medicare Advantage with at least 2 months of pre-COVID data means the study population includes people who have relatively good access to medical care. The authors do not report what % had commercial insurance, but those people would have been healthy (and young enough) to have employment. How representative is this of the overall population?
 - What about care people may have received in other locations, e.g., out of network?
 - The study time is limited to largely pre-vaccination stages of the pandemic, and during early variants, when monoclonals were available and used more widely – would these findings be generalizable or informative now?

**R5:** Thank you for your observations. We have expanded our limitations to include dataset limitations, potential exclusion of out of network and uninsured patients, the average age of our population and the stage of the pandemic from which our patient cohort was obtained**.** data.

**C6:** Line 181 – There is an interesting but unfounded (by reproducible studies) assumption that patients are ‘merely anxious or depressed’ after SARS-CoV-2 infection, implying they should be able to use CBT or positive self-talk to resolve their symptoms. In an effort to discourage these and other similar implications, please remove the phrase “decline in mental health” from line 181 and instead simply report the numbers.

**R6:** This has been removed.

**Reviewer #2:**

**C7:** This study provides evidence of healthcare utilization (HCU) trends of COVID-19 patients 9 months post-diagnosis using a sample of the U.S. COVID-19 patient population. The cohort creation process is transparent and the paper is well written. However, the current analysis is not sufficient to merit publication at PLOS ONE. First, it is not entirely clear what is the biggest contribution of the study. Given that there are other studies that extend the time horizon of long COVID HCU to twelve months (Roth et al., 2022), the authors should argue how these results may be more informative to other longitudinal studies of HCU and long COVID on the basis of the quality of their analysis, as opposed to the long time span of the COVID-19 post-diagnosis period only. It is not surprising that HCU rises post-diagnosis; the authors could also try to timestamp when this increased HCU subsides for different age groups (Koumpias et al., 2022).
 The authors should consider these easily executable revisions below to enhance the rigor of the analytical approach.

**R7:** Thank you for your comment. Our study has four main findings: 1 Increase in HCU post-discharge home in a cohort of patients studied pre-and post-COVID-19 hospitalization. Current studies include ambulatory patients and or patients discharged to multiple settings (including home) when they evaluate post-discharge HCU. 2: Women have increase in post discharge HCU in some of the outcomes but not others. 3. The post-discharge HCU may be primarily driven by patients who received care in the ICU.4. The significant increase in visits to different medical providers but specifically to pulmonary medicine. To strengthen our findings, we have moved from a descriptive approach of HCU to a direct comparison of HCU pre and post-COVId-19 hospitalization, added a sensitivity analysis to compare HCU by sex and by ICU and provide rate ratios and p values to show statistically significant values.
**C8:** The biggest concern is the lack of any account of patient insurance status. The confluence of Medicare-eligible and Medicare non-eligible population in the study cohort leads to overestimation of the influence of a COVID-19 diagnosis on HCU because the latter group (<65years old) is less likely to engage in HCU due to relatively higher costs they may face. Therefore, the authors should report HCU separately for commercially insured (<65yrs old) and Medicare/Medicare Advantage patients.

**R8:** Thank you for your comment. We have now clearly identified the limitations of our dataset and cohort in the manuscript. Please see the limitations section in the discussion. This data set did not have patients with Medicare Advantage.

Additionally, our contract to access CDM raw data ended on 6/30/2023, we cannot provide further information regarding the percentage of patients that were commercially insured vs those with Medicare.

Below is the table for the number of patients below the age of 65

| **Age** | **Frequency** | **Percent** | **Cumulative** **Frequency** | **Cumulative** **Percent** |
| --- | --- | --- | --- | --- |
| **<65** | 13764 | 21.79 | 13764 | 21.79 |
| **>=65** | 49397 | 78.21 | 63161 | 100.00 |

**C9: It** is also mentioned that this is a descriptive analysis; yet no statistical tests are being used, whatsoever. At a minimum, the authors should report whether pre-, post-diagnosis levels are statistically different using t-tests. For instance, the first sentence of the introduction reads: "In our nationally-representative study of patients discharged home after a COVID-19 hospitalization, we found that HCU remained significantly high 9 months after discharge from index hospitalization." It appears highly likely that the increase in HCU is detectable at conventional levels of statistical significance, too. After conducting the statistical analysis, this statement could be revised to explicitly state whether increased HCU remained higher in a statistically significant way.

**R9:** Thank you for your comment. We have taken the advice of the reviewer and performed statistical analyses. We now compare the pre and post hospitalization HCU, provide percentage change, rate ratios with confidence intervals and p values. Please see results section in the manuscript and tables2-3, and S5-6.

**C10:** Another concern is that HCU is driven by intensity of hospitalization which varies in a way that the authors do not sufficiently account for. Separating the results by specialty is certainly a step in that direction. It would be most informative to show how the trends of post-diagnosis HCU for different subgroups of varying hospitalization intensity. In fact, the authors should consider shedding light to any differential HCU responses by LOS. (e.g. by LOS quartiles). This would make a genuine contribution by further illustrating post-discharge differences in HCU based on severity of COVID-19 infection. However, the authors do not mention changes in patient LOS until page 9. Given that there is relatively less evidence on LOS changes than HCU changes following COVID-19 diagnosis, this novel finding should be discussed earlier if not employed as another variable in cross-tabulations of HCU pre- and post-diagnosis.
**R10:** Thank you for your comment. We have performed sensitivity analysis and found differences in HCU in women compared to men and in patients who received care in the ICU vs those who didn’t. In addition, our contract to access CDM raw data was ended on 6/30/2023, so we cannot perform further analysis of the data.

**C11:** Another source of measurement error causing underestimation of the association of COVID-19 with post-diagnosis HCU is due to sample attrition from death or loss of insurance. To examine the sensitivity of their findings to this potential issue, the authors should use a balanced panel of patients who contribute person-days both pre-diagnosis and during the third post-diagnosis time interval (6-9) as a supplementary robustness check.
Related, the authors should explore whether information regarding COVID-19 diagnosis is available in the secondary and subsequent diagnosis lines and to what extent this leads to significant undercount of COVID-19 patients. It would be very helpful to complement the results a discussion of the frequency of COVID-19 diagnosis non-primary line reporting.

**R11:** Thank you for your comment. We cannot determine the post-discharge mortality rate in our cohort; however, with an estimated 7.8% all-cause post-discharge mortality rate reported in the literature (Ramzi ZS. Hospital readmissions and post-discharge all-cause mortality in COVID-19 recovered patients; A systematic review and meta-analysis. *Am J Emerg Med*. 2022;51:267-279. doi:10.1016/J.AJEM.2021.10.059), we know most patients discharged from a COVID-19 hospitalization are alive one year post discharge. To address this limitation, our cohort only included patients enrolled in insurance/database until the end of the study period. Therefore, each patient contributed person-days until death, loss of insurance eligibility, or the end of the 9-month follow-up period. Please see the limitations section of the main manuscript.

Please see the following table. Our statistics team performed a balanced analysis and noted significant overall and were significant in the 3- or 6-months balanced cohorts. The 3-month interval included almost all of the patients, 6-month captured about half and 9-month about 20%. The 6- and 9-month intervals yielded similar, consistent results.

|  | **Pre-covid, mean (SD)** | **Post-covid, mean (SD)** | **p-value*** |
| --- | --- | --- | --- |
| **Inpatient admission (los)** | 1.78 (7.53) | 2.53 (8.76) | <.0001 |
| **ED visits** | 0.34 (0.78) | 0.90 (1.93) | <.0001 |
| **Rehab visits** | 0.04 (0.24) | 0.14 (0.50) | <.0001 |
| **Office visits** | 1.72 (2.50) | 8.00 (7.93) | <.0001 |
| **Telemedicine visits** | 0.17 (0.56) | 0.50 (1.63) | <.0001 |

*p-value from paired ttest or non-parametric Wilcoxon signed rank test as appropriate

Also, our cohort selection focused on patients discharged home from a hospitalization with a primary diagnosis of COVID-19. We do agree with the reviewer that it would be interesting to know if there is a significant undercount of patients hospitalized with COVID-19, the above data would not affect our cohort selection process. In addition, our contract to access CDM raw data was ended on 6/30/2023, so we cannot perform further analysis of the data.

Additionally, we excluded 26.4% (42,305 patients) of patients from our cohort because they do not have COVID-19 as their primary diagnosis. Please see Fig 1.

Lastly, Optum data is proprietary (and we have purchased access to it under contract), therefore, we are not permitted to share the data.

**C12:** Re: Data Availability - Unsure whether data is available after all given that this is the proprietary OptumInsights de-identified Clinformatics Data Mart. This may need to be corrected.
Finally, are the results robust to the inclusion of the outcome in its raw form, measured in levels? It would be useful to show whether the transformation to person-days has any influence on the results.
**R12:**

Thank you for the comment. We have access to the claim-level data for us to perform the analyses. We use procedure and service codes to identify the HCU pre and post Covid hospitalization per person. The outcomes are then aggregated to generate count data.
We discussed this within our group and thought that since each person has different follow-up days, interpreting the data would be more meaningful if compared to the HCU after adjusting by person-days.

**C13:**
A quick literature review identified the following journal articles pertinent to this study:
References:
- Koumpias AM, Schwartzman D, Fleming O. Long-haul COVID: healthcare utilization and medical expenditures 6 months post-diagnosis. BMC Health Services Research. 2022 Aug 8;22(1):1010.
 - Roth SE, Govier DJ, Marsi K, Cohen-Cline H. Differences in outpatient health care utilization 12 months after COVID-19 infection by race/ethnicity and community social vulnerability. International Journal of Environmental Research and Public Health. 2022 Mar 15;19(6):3481.
 - Zhou X, Andes LJ, Rolka DB, Imperatore G. Changes in health care utilization among Medicare beneficiaries with diabetes two years into the COVID-19 pandemic. Ajpm Focus. 2023 Jun 1:100117.

**R13:**
Thank you for these suggestions. We have incorporated the first 2 papers into the paper. The findings of the third paper were very interesting however the population was not focused on post-hospitalization healthcare utilization like our population.
