# Supplementary material for: Healthcare utilization 9 months pre- and post- COVID-19 hospitalization among patients discharged alive
Source: PLoS One. 2024 Jun 20;19(6):e0303509. doi: 10.1371/journal.pone.0303509 (PMC11189225; doi:10.1371/journal.pone.0303509)
Supplement: Attachment — Submitted filename: Response to Reviewers - 11-17-23.docx [file pone.0303509.s003.docx]

**Reviewers' comments:**

**Reviewer's Responses to Questions**

**Comments to the Author**

**1. If the authors have adequately addressed your comments raised in a previous round of review and you feel that this manuscript is now acceptable for publication, you may indicate that here to bypass the “Comments to the Author” section, enter your conflict of interest statement in the “Confidential to Editor” section, and submit your "Accept" recommendation.**

Reviewer #1: (No Response)

Reviewer #2: All comments have been addressed

2. Is the manuscript technically sound, and do the data support the conclusions?

The manuscript must describe a technically sound piece of scientific research with data that supports the conclusions. Experiments must have been conducted rigorously, with appropriate controls, replication, and sample sizes. The conclusions must be drawn appropriately based on the data presented.

Reviewer #1: Partly

Reviewer #2: Yes

3. Has the statistical analysis been performed appropriately and rigorously?

Reviewer #1: No

Reviewer #2: Yes

4. Have the authors made all data underlying the findings in their manuscript fully available?

The [PLOS Data policy](https://nam11.safelinks.protection.outlook.com/?url=http%3A%2F%2Fwww.plosone.org%2Fstatic%2Fpolicies.action%23sharing&data=05%7C01%7Cmfzaidan%40utmb.edu%7Cb0984e793af7459909b508dbce1a69bf%7C7bef256d85db4526a72d31aea2546852%7C0%7C0%7C638330385088117212%7CUnknown%7CTWFpbGZsb3d8eyJWIjoiMC4wLjAwMDAiLCJQIjoiV2luMzIiLCJBTiI6Ik1haWwiLCJXVCI6Mn0%3D%7C3000%7C%7C%7C&sdata=oEwBgMXHVtCeL7xjX3Ky8DQvF5J1GZQyVp4XbvG6qsc%3D&reserved=0) requires authors to make all data underlying the findings described in their manuscript fully available without restriction, with rare exception (please refer to the Data Availability Statement in the manuscript PDF file). The data should be provided as part of the manuscript or its supporting information, or deposited to a public repository. For example, in addition to summary statistics, the data points behind means, medians and variance measures should be available. If there are restrictions on publicly sharing data—e.g. participant privacy or use of data from a third party—those must be specified.

Reviewer #1: No

Reviewer #2: No

5. Is the manuscript presented in an intelligible fashion and written in standard English?

PLOS ONE does not copyedit accepted manuscripts, so the language in submitted articles must be clear, correct, and unambiguous. Any typographical or grammatical errors should be corrected at revision, so please note any specific errors here.

Reviewer #1: Yes

Reviewer #2: Yes

6. Review Comments to the Author

Please use the space provided to explain your answers to the questions above. You may also include additional comments for the author, including concerns about dual publication, research ethics, or publication ethics. (Please upload your review as an attachment if it exceeds 20,000 characters)

**Reviewer #1:**

The authors have responded to some questions and comments to the best of their ability, and comparison of 9 months pre- vs. post HCU hospitalization for COVID was added, which his helpful. However, there remain significant limitations within the available data that perhaps cannot be addressed. Overall, however, it is not surprising that people who were hospitalized required more health care after hospitalization and they did poorly in general, even among those who were only eligible because they survived at least a year after hospital discharge.

**R1:**
**One key question that this study cannot answer is whether or to what degree hospitalization for COVID may differ from other causes of hospitalization.**

**A1:**

Thank you for the criticism. Our main purpose of this article was to provide a descriptive analysis of health care utilization of patients post hospitalization for COVID pneumonia. We did not intend to compare how hospitalization for COVID pneumonia may differ from other pneumonias or other causes. Other published works have made such comparisons, such as these 2 articles.

COVID pneumonia hospitalization had increased pulmonary shunts that is not commonly seen in other types of pneumonia. (Novelli et al) Another article noted that patients with COVID pneumonia who required mechanical ventilation had longer times to liberation from mechanical ventilation. (Nolley et al).

Novelli, Malandrino, Balbi, et al. Shunt fraction and radiological involvement in Covid-19 related Acute Respiratory Failure. European Respiratory Journal Sep 2023, 62 (suppl 67) PA5105; DOI: 10.1183/13993003.congress-2023.PA5105

Nolley EP, Sahetya SK, Hochberg CH, et al. Outcomes Among Mechanically Ventilated Patients With Severe Pneumonia and Acute Hypoxemic Respiratory Failure From SARS-CoV-2 and Other Etiologies. JAMA Netw Open. 2023;6(1):e2250401. doi:10.1001/jamanetworkopen.2022.50401

**R2:**
**Can the authors clearly explain what this study adds beyond a prior publication (**[**https://www.ncbi.nlm.nih.gov/pmc/articles/PMC9548925/**](https://www.ncbi.nlm.nih.gov/pmc/articles/PMC9548925/)**) how it adds materially to our understanding of health care use post hospitalization for COVID (specifically, compared to other reasons for hospitalization).**

**A2:**
Thank you for pointing out this prior publication. This was our poster publication that was presented at the CHEST conference 2022 and published online. Since this abstract, we have performed a statistical analysis and sensitivity analysis on healthcare utilization post discharge the index hospitalization.

Previous studies compared HCU between patients with and without COVID.

In this article, we did within subject comparison (before and after COVID for each patient). We also compared the within subject changes by gender and ICU. We did not intend to compare how hospitalization for COVID pneumonia may differ from other pneumonias or other causes.

**R3:**
**Otherwise, I have the following 3 additional comments/questions:**

Please provide a brief description of the kinds of patients who would be included in Optum’s Clinformatics Data Mart – what % of the US population are generally included?

Are these people generally of similar, worse, or better health than the general population?

What kinds of medical centers contribute data, e.g., typically community vs. academic? What % are rural?

Readers have a general idea of, for example, the patient population and generalizability for VHA studies, but that is not the case here. For provide a similar summary for patients in this cohort.

**A3:**

- Optum’s Clinformatics Data Mart contains claims from enrollees with commercial insurance or Medicare Advantage Plans. The database comprises of over 63 million unique enrollees in the United States from all states.
- The majority (>= 95%) of the enrollees in Optum’s CDM have commercial insurance.
- Traditional Medicare or Medicaid enrollees are not included in the database, so it is likely that the dataset is less representative of the older or low socioeconomic status population.
- Because the data in Optum’s CDM come from insurance claims, there are no limitations on the type of medical center that can contribute data (e.g., rural / urban or academic/community).
- When a CDM patient files a claim with their insurance, the data are captured, regardless of the medical center/office he visited.

We have added these points to the Data Source paragraph in the methods section

**R4:**
Page 7 line 127 – Please clarify that this was the primary admission diagnosis (In Figure 1, I see that this appears to be a hospital admission diagnosis, is that correct?)

**A4:**
Yes. This was the primary admission diagnosis for the hospitalization.

**R5:**
In the discussion/limitations, please include the risk of introducing survivor bias by requiring that people have at least 12 months of follow-up. Why not allow variable follow-up and simply account for it in analysis, and not risk survivor bias?

**A5:**
Thank you for your comment. To address the concern of survivor bias, we required participants to have at least 12 months of data prior to their primary COVID-related hospitalization for accurate comorbidity assessment and utilized a person-days approach to accommodate variable follow-up durations. We did not require follow-up AFTER hospitalization. We used person-days to capture to allow for variable follow up and limit survivor bias.

**R5:**

What are the test characteristics for the ICD code for accurate case identification? (e.g., sensitivity, specificity, PPV, NPV)

**A5:**

While there are no published studies on ICD code accuracy using Optum’s CDM specifically, a study (Kadri et. Al) using the same ICD-codes for identification of COVID-19 disease in another insurance claims database found the following: "sensitivity was 98.01% (95% CI, 97.62%-98.39%), the specificity was 99.04% (95% CI, 98.95%-99.13%), the positive predictive value was 91.52% (95% CI, 90.77%-92.27%), and the negative predictive value was 99.79% (95% CI, 99.75%-99.83%).”

Kadri SS, Gundrum J, Warner S, et al. Uptake and Accuracy of the Diagnosis Code for COVID-19 Among US Hospitalizations. *JAMA.* 2020;324(24):2553–2554. doi:10.1001/jama.2020.20323

**R6:**
I initially misread Figure to read that 63,161 people were excluded due to loss of insurance prior to hospital discharge. Please restructure Figure 1 to list the N excluded (%), similar to reporting for clinical trials.

**A6:**
Thank you for the suggestion. We have made this change.
Please see the new Figure 1

**R7:**
Table 1: Lack of insurance information is an important limitation. How were the comorbid conditions identified (i.e., in a supplement, include ICD codes, etc)

**A7:**
All patients had insurance under Optum’s insurance contractor (large national commercial insurance or Medicare Advantage). Over 95% had commercial insurance and a little over 4% had Medicare advantage (we unfortunately do not have more specific information on insurance for each patient). We required all patients covered by Optum insurance contracts in the 12 months before hospitalization in order to identify comorbidity.

Additonally, we have added this table in the supplement section

| **Disease** | **ICD-10 codes** |
| --- | --- |
| COVID-19 | U07.1 |
| Diabetes | E10.xx, E11.xx, E13.xx |
| Hypertension | I10.x, I11.xx, I12.xx, I13.xx I15.xx, I67.4 |
| Asthma | J45.xx |
| COPD | J41.8, J42.xx, J43.xx, J44.xx |
| CKD | N18.9 |
| ESRD | N18.6 |
| Stroke | I63.xx, I64.xx, I69.3x, G45.9 |
| Heart disease | I09.9, I11.0, I13.xx, I25.5, I42.xx, I43.xx, I50.xx |
| Cancer | C00.x – C96.x |
| CAD | I25.1X |
| Liver disease | K70 – K74, K76.xx |

**R8:**
Table 2: Please move the units for each measure of HCU to the row label to make them more clear and please provide more explanation of the measures of health care utilization. E.g., “ED visits per 10,000 person-days”.

**A8:**
Thank you for this suggestion. Completed.

**R9:**
Are the reported pre- vs. post-hospitalization HCU reflective of the mean or median or some other measure?

**A9:**
We report healthcare use (e.g. number of visits) as incidence per 10,000 person – days. It is an epidemiologic measure, which accounts for the actual time each participant contributes to the study.
We mention this on lines 147-150 and each table has this mentioned at the bottom.

**R10:**
Please include the appropriate measure of variability around that reported summary (e.g., SD, IQR, etc). Is hospital LOS per capita as well?

**A10:**

There is no measure of variability for incidence, other than the reported risk ratio when comparing two incidence rates. We reported LOS as total days of inpatient stay per 10,000 person-days.

**R11:**
Why was 9 months before and after chosen? Why not a year? (A year would be easier to compare to other information, e.g., the baseline proportion of people with an ED visit per year, to assess generalizability)

**A11:**
During the time of our initial data acquisition, we didn't have data for 12 months post-COVID hospitalization.

**R12:**
Please confirm that post hospitalization HCU measures do not include the index hospitalization.

**A12:**
This is correct. All the HCU measures DID NOT include the index hospitalization.
**R13:**
Tables 1, 2, and 3 are currently in the Methods section, but shouldn’t they be referred to and located in the Results section?

**A13:**
Thank you for the suggestion, the tables have been moved to the results section.

**R14:**
Why might people who were in the ICU have shorter length of stay? (How does that translate into a RR>1? Am I interpreting that result incorrectly?)

**A14:**
Thank you for your comment. We found that increase of covid HCU from pre to post period in patients with ICU stays was larger than those without, hence the RR>1. Those patients with ICU stays during COVID hospitalization had much large increase of length of stay than those without.

**Discussion:**
**R15:**
For people in other countries, please provide some context for health care utilization. In general, how much might an outpatient visit cost, out of pocket?

**A15:**
Ambulatory care, visit complexity is stratified from Level 1 for minor issues, costing an average of $46, to Level 5 for the most severe cases at $182 per visit. Level 3, the median for common visits, averages $90. The overall outpatient visit cost in 2018 averaged $105.

From 2008 to 2018, the cost for Level 1 visits rose by $15 (52%), while Level 5 visits increased by $49 (37%). Level 3 visits, the most frequent, saw a $20 rise (29%), indicating a notable trend in healthcare expenditure growth.

"How costly are common health services in the United States?" Peterson-KFF Health System Tracker. https://www.healthsystemtracker.org/chart-collection/how-costly-are-common-health-services-in-the-united-states/#item-start. Accessed November 7, 2023.

**R16:**
What % of a hospitalization might patients have to pay out of pocket, and approximately how much might that be in absolute USD? This provides context for what might drive patient behavior, specifically health-seeking behaviors. Might some patients avoid outpatient care if it cost $25/visit, while an ED visit or in-patient hospitalization might have $0 out of pocket costs?

**A16:**
Hospitalization costs incur varied out-of-pocket expenses, influenced by insurance coverage. In 2020, average out-of-pocket costs for COVID-19 hospitalizations were approximately $1,653 under traditional plans and $1,961 for consumer-driven plans. A related study indicated a median cost for privately insured patients ranging from $59 to $842 due to COVID-19.

"Assessment of Out-of-Pocket Spending for COVID-19 Hospitalizations in the US in 2020." JAMA Network. https://jamanetwork.com/journals/jama/fullarticle/2780416. Accessed November 7, 2023.

On a broader note, U.S. employees paid an average of $1,763 out-of-pocket before reaching their insurance deductibles last year. Such costs are substantial and may affect decisions regarding follow-up outpatient care, in contrast to some plans covering emergency or inpatient services fully, thereby incurring no out-of-pocket expenses

"Average out-of-pocket healthcare costs." Bankrate. https://www.bankrate.com/insurance/health/average-out-of-pocket-healthcare-costs/

**R17:**
Is it possible to include any information about why patients were re-hospitalized? E.g., admission for ACS, neurological, PNA, dyspnea, or other causes?

**A17:**
Unfortunately, our contract of the Optum data set was expired on June 2023 and this is not possible.

**R18:**
For consistency, I suggest using the terms male and female throughout, since sex was likely the measured variable in available data, not gender.

**A18:**
Thank you for the suggestions. We have made the appropriate changes.

**Reviewer #2: (No Response)**

7. PLOS authors have the option to publish the peer review history of their article ([what does this mean?](https://nam11.safelinks.protection.outlook.com/?url=https%3A%2F%2Fjournals.plos.org%2Fplosone%2Fs%2Feditorial-and-peer-review-process%23loc-peer-review-history&data=05%7C01%7Cmfzaidan%40utmb.edu%7Cb0984e793af7459909b508dbce1a69bf%7C7bef256d85db4526a72d31aea2546852%7C0%7C0%7C638330385088117212%7CUnknown%7CTWFpbGZsb3d8eyJWIjoiMC4wLjAwMDAiLCJQIjoiV2luMzIiLCJBTiI6Ik1haWwiLCJXVCI6Mn0%3D%7C3000%7C%7C%7C&sdata=ATcwLag2VDBTae7RJfVWmhE0QLe%2BPIxvt%2Fay83%2F7hho%3D&reserved=0)). If published, this will include your full peer review and any attached files.

If you choose “no”, your identity will remain anonymous but your review may still be made public.

Do you want your identity to be public for this peer review? For information about this choice, including consent withdrawal, please see our [Privacy Policy](https://nam11.safelinks.protection.outlook.com/?url=https%3A%2F%2Fwww.plos.org%2Fprivacy-policy&data=05%7C01%7Cmfzaidan%40utmb.edu%7Cb0984e793af7459909b508dbce1a69bf%7C7bef256d85db4526a72d31aea2546852%7C0%7C0%7C638330385088117212%7CUnknown%7CTWFpbGZsb3d8eyJWIjoiMC4wLjAwMDAiLCJQIjoiV2luMzIiLCJBTiI6Ik1haWwiLCJXVCI6Mn0%3D%7C3000%7C%7C%7C&sdata=9k6YpE7ZkTxWeHIjZBuT4RyKX7hTLSEJLS6No6d7loU%3D&reserved=0).

Reviewer #1: No

Reviewer #2: No

[NOTE: If reviewer comments were submitted as an attachment file, they will be attached to this email and accessible via the submission site. Please log into your account, locate the manuscript record, and check for the action link "View Attachments". If this link does not appear, there are no attachment files.]

While revising your submission, please upload your figure files to the Preflight Analysis and Conversion Engine (PACE) digital diagnostic tool, [https://pacev2.apexcovantage.com/](https://nam11.safelinks.protection.outlook.com/?url=https%3A%2F%2Fpacev2.apexcovantage.com%2F&data=05%7C01%7Cmfzaidan%40utmb.edu%7Cb0984e793af7459909b508dbce1a69bf%7C7bef256d85db4526a72d31aea2546852%7C0%7C0%7C638330385088117212%7CUnknown%7CTWFpbGZsb3d8eyJWIjoiMC4wLjAwMDAiLCJQIjoiV2luMzIiLCJBTiI6Ik1haWwiLCJXVCI6Mn0%3D%7C3000%7C%7C%7C&sdata=T6hH9npsZv0Ck4tXUKEuoT%2FTKZRp9H29De%2BNdXvE6ls%3D&reserved=0). PACE helps ensure that figures meet PLOS requirements. To use PACE, you must first register as a user. Registration is free. Then, login and navigate to the UPLOAD tab, where you will find detailed instructions on how to use the tool. If you encounter any issues or have any questions when using PACE, please email PLOS at [figures@plos.org](mailto:figures@plos.org). Please note that Supporting Information files do not need this step.

*In compliance with data protection regulations, you may request that we remove your personal registration details at any time.* [*(Remove my information/details)*](https://nam11.safelinks.protection.outlook.com/?url=https%3A%2F%2Fwww.editorialmanager.com%2Fpone%2Flogin.asp%3Fa%3Dr&data=05%7C01%7Cmfzaidan%40utmb.edu%7Cb0984e793af7459909b508dbce1a69bf%7C7bef256d85db4526a72d31aea2546852%7C0%7C0%7C638330385088117212%7CUnknown%7CTWFpbGZsb3d8eyJWIjoiMC4wLjAwMDAiLCJQIjoiV2luMzIiLCJBTiI6Ik1haWwiLCJXVCI6Mn0%3D%7C3000%7C%7C%7C&sdata=uVNRcxt6XZm%2BNj2yUSdiJkVC0TxiYrmd4Li8nj4ceLs%3D&reserved=0)*. Please contact the publication office if you have any questions.*
