# Supplementary material for: Healthcare utilization 9 months pre- and post- COVID-19 hospitalization among patients discharged alive
Source: PLoS One. 2024 Jun 20;19(6):e0303509. doi: 10.1371/journal.pone.0303509 (PMC11189225; doi:10.1371/journal.pone.0303509)
Supplement: Attachment — Submitted filename: Response to reviewers FINAL MZ 3-27 .docx [file pone.0303509.s004.docx]

Reviewer #3:

**Comment:** The tables should be referred to in the results & not the methods. They also have numerous subscripts within the title of the tables that are unnecessary. Just need a table legend without the addition of subscripts within the title (Minor point).

Response: Thank you for your comment. All tables references have been removed from the methods. Tables are referenced in the results section. Also, subscripts from the titles have been removed and the table legends summarized.

**Comment:** I am slightly confused about the inclusion/exclusion criteria. In the "Cohort Selection" section of the methods, the authors state that "Our samples included all adults hospitalized with primary diagnosis of COVID-19 who were discharged home (with or without HOME HEALTH care)..." However, in Figure 1 they state that "Not discharged home or with HOME HEALTH" was part of the exclusion criteria. This needs to be clarified, were those with HOME HEALTH included of excluded. This may have an impact on the amount of HCU. If HOME HEALTH participants were included then this would likely have an impact on HCU.
**Response:** Thank you for your comment. We agree with the reviewer. The way figure one was written lead to potential confusion. To clarify, our study only included patients who were discharged home (with or without home health). So, if a patient was not discharged home, then it was excluded. Figure one has been edited to clarify this point.

Comment: Similarly, another potentially observation that could inform future pandemic preparedness would be analysis on whether particular co-morbidities were associated with increased HCU post-COVID discharge.
Response: PLEASE RESPOND

**Comment:** Please define OFFICE (mentioned in the results (e.g. p13; line 232, p14; line 248) which was not described within the results.
**Response:** Thank you for your comment. We have added the definition of office in the methods section. A copy of the paragraph follows “. Based on billing information, we examined outpatient visits (from here on out referred to as office visits) to primary care providers, including family medicine, internal medicine, and nurse practitioner visits, and certain sub-specialties, such as cardiology, pulmonary medicine, endocrinology, neurology, physical medicine and rehabilitation, psychiatry, and other mental health professionals”

Comment: A limitation of this work is perhaps the limited ability to extrapolate these findings to a larger cohort (throughout the USA), given that this cohort is likely to have a higher socioeconomic status (given the degree of insurance cover) and are of older age (72 years). If the authors were to divide the cohort by age (in a sub analysis) this would add more weight to generalizing to a larger population.

**Response**: Thank you for your comment. Socioeconomic status has been added as a limitation. Please see discussion section under limitations.

Please see the table below. We have made a table that divides the cohort by age. This has been added to the supplemental tables.

| **Age** | **<65** | | | **>=65** | | | **Rate ratio post-covid >=65 vs<65** |
| --- | --- | --- | --- | --- | --- | --- | --- |
|  | **Pre-covid** | **Post covid** | **Percent change** | **Pre-covid** | **Post covid** | **Percent change** |  |
|  | **-0-9 months** | **+0-9 months** |  | **-0-9 months** | **+0-9 months** |  |  |
| **Inpatient admission (los)** | 33.95 | 139.66 | 311.32 | 45.31 | 173.94 | 283.86 | 1.25 (1.23-1.26) |
| **Inpatient visits** | 5.67 | 19.24 | 239.40 | 7.41 | 23.95 | 223.07 | 1.24 (1.20-1.29) |
| **ED visits** | 25.33 | 49.81 | 96.69 | 21.30 | 48.16 | 126.12 | 0.97 (0.94-0.99) |
| **Rehab visits** | 1.10 | 2.94 | 167.46 | 3.62 | 9.18 | 153.79 | 3.12 (2.85-3.44) |
| **Office visits** | 155.87 | 444.66 | 185.28 | 159.87 | 370.97 | 132.04 | 0.83 (0.82-.84) |
| **Telemedicine visits** | 8.67 | 22.45 | 158.87 | 12.09 | 26.17 | 116.39 | 1.17 (1.12-1.21) |
